# Response to the letter to the editor: Sociodemographic considerations regarding the European Portuguese Validation of the Diabetes Distress Scale (DDS-17)

**DOI:** 10.20945/2359-4292-2026-0099

**Published:** 2026-08-23

**Authors:** Maria Inês Serpa Paz Teixeira de Melo, Diana Falcão, Dina Isabel Campos, Mariana M. dos Santos, Jorge Hernâni-Eusébio, Anabela Barreto Silva

**Affiliations:** 1 School of Medicine, University of Minho, Braga, Portugal; 2 Family Health Unit of Minho, Braga Local Health Unit, Braga, Portugal; 3 Gualtar Family Health Unit, Braga Local Health Unit, Braga, Portugal; 4 Life and Health Sciences Research Institute (ICVS), School of Medicine, University of Minho Campus de Gualtar, Braga, Portugal; 5 Trofa Saúde Braga Sul Hospital, BRAGAFirst – Practice Based Research Network, Braga Local Health Unit, Braga, Portugal; 6 BRAGAFirst – Practice Based Research Network, Braga Local Health Unit, Braga, Portugal; 7 Sá de Miranda Family Health Unit, Braga Local Health Unit, Vila Verde, Portugal

Dear Editor,

We would like to express our gratitude for the insightful and pertinent comments regarding our article “European Portuguese Validation of the Diabetes Distress Scale (DDS-17)”(^1^). The questions raised concerning the sociodemographic characteristics of our sample, specifically age and educational level, significantly enrich the discussion on the clinical applicability and robustness of this scale within the Portuguese population (^2^).

Regarding the impact of age on diabetes distress, we acknowledge that literature, such as the study by Almeida et al. cited by the commentators, suggests a reduction in overall perceived stress in older adulthood (^3^). However, it is crucial to distinguish general life stress from diabetes distress, which measures the distinct emotional burden inherently tied to managing a chronic condition. Furthermore, the mean age of our cohort (69.55 ± 12.55 years) is not an artifact of selection bias, but rather a faithful reflection of the epidemiological reality of Type 2 Diabetes (T2D) in Portugal, where the highest prevalence of the disease is concentrated precisely within the 60 to 79 age group (^4^). In these older patients, diabetes management often coexists with multiple comorbidities and age-related functional decline, which may, conversely, exacerbate disease-specific distress. Therefore, while the low global scores in our sample warrant cautious interpretation, they represent the actual clinical presentation of the target population.

In relation to educational attainment, the high prevalence of low formal education (53.0% with four years or less) also accurately reflects the historical and socio-educational context of this specific demographic in Portugal. For generations born until 1966, mandatory school attendance was legally limited to just four years. Consequently, any representative sample of older Portuguese T2D patients will naturally exhibit this characteristic. To actively mitigate the risks of non-response, reading comprehension difficulties, or cognitive fatigue highlighted by the commentators, the administration of the questionnaire in our study included dedicated assistance from healthcare professionals. They provided assisted reading and clarified individual items whenever a need was identified. This methodological safeguard minimized literacy barriers, ensuring that the scores reflected the patients' genuine emotional experiences rather than comprehension errors. The strong internal consistency and construct validity demonstrated in our paper further corroborate that the scale remained psychometrically robust within this population.

Finally, addressing the inquiry regarding subgroup analyses, this specific evaluation was not performed in the original article, as it fell outside the scope of this baseline psychometric validation study. However, we are pleased to share that this exact line of investigation is already underway. A follow-up study with a considerably larger and more diverse sample is currently being conducted, which will provide the statistical power necessary to thoroughly clarify the variability of distress across different age groups and educational levels, as well as to investigate the risk of false negatives.

We entirely endorse the commentators' recommendation that the clinical implementation of the DDS-17 in Portugal should be coupled with assisted reading strategies in populations with lower literacy, a practice we proactively adopted in our study design. We thank the commentators once again for their valuable contribution, which reinforces the importance of continuous debate to optimize comprehensive diabetes care.

## Data Availability

datasets related to this article will be available upon request to the corresponding author.
